# Credit to Whom Credit Is Due

**Published:** 1883-12

**Authors:** 


### CREDIT TO WHOM CREDIT IS DUE.

The article entitled "Dental Periostitis ; The Grindstone Cure," in the October number of this journal, was, through a misunderstanding, printed as though written expressly for us. We were indebted for it to the courtesy of Dr. J. Edw. Line, editor of *The Odontographic Journal,* who sent us a copy in advance of his own publication of it. As the INDEPENDENT PRACTITIONER was out a few days before the *Odontographic,* he had the gratification of seeing it in another journal, without the proper credit, before its appearance where it properly belonged; a joke which he perhaps did not fully appreciate. Our excuse is that we supposed we were *personally* indebted to our friend Line, and not to the editor of the best dental quarterly published.

